# Gene expression level: is it an important factor in codon optimization for overexpression of recombinant proteins?

**DOI:** 10.1186/1753-6561-8-S4-P224

**Published:** 2014-10-01

**Authors:** Juliana da Costa Ramos, Jennifer Malaguez Oliveira, Carlos Frederico Ceccon Lanes, Marcio de Azevedo Figueiredo, Luis Fernando Marins

**Affiliations:** 1 Instituto de Ciências Biológicas, Universidade Federal do Rio Grande, FURG, Rio Grande, RS, Brazil; 2 Insituto de Oceanografia, Universidade Federal do Rio Grande, FURG, Rio Grande, RS, Brazil

## Background

Microalgae are becoming a viable model for the expression of recombinant proteins. However, suitable levels of expression for commercial production have not yet been obtained. One way to increase the heterologous protein production is the codon optimization of the target gene so that it presents the preferential codons used by the host organism according to its own pool of transfer RNA (tRNA). For codon optimization the Codon Usage Database (CUD; http://www.kazusa.or.jp/codon) has been used. The database provides codon frequency tables based on gene transcripts, regardless of their level of expression. Therefore, the aim of this study was to determine if there are changes in the frequency of codons used when taking into account the level of expression of genes, considering the 50 most expressed genes and 50 least expressed genes from the microalgae *Chlamydomonas reinhardtii*.

## Methods

The transcriptome data, published by Lv et al. (2013), was used to select the genes according to their expression level. The 50 most expressed transcripts (RPKM>2900) were selected from the four stages of growth of *C. reinhardtii*: Log (LP), stationary (SP), lipid accumulation (LAP), and cellular decline (CDP). As a negative control, we selected the 50 least expressed transcripts (RPKM<15) from the LP growth phase. The sequences of the selected genes were obtained from the website http://genome.jgi-psf.org/Chlre4/. The codon frequency tables were created using the tool Gene to Codon Usage (http://www.entelechon.com/2008/10/gene-to-codon-usage/). Tables, including the one provided by CUD, were compared via the UPGMA method (http://genomes.urv.cat/UPGMA) and by the statistical test of chi-square using the software R.

## Results and conclusion

The tables created with the 50 most expressed genes in the four stages of growth of the microalgae were not different by analysis of UPGMA. Thus, the LP stage table was used for all other comparisons. The UPGMA method distinguished the three tables. The main difference among the tables was observed in the AAG codon, with a frequency of 98.6 per thousand in the 50 most expressed genes table, compared with 43.3 per thousand from the CUD, and 28.0 from the negative control. Moreover, codons with zero frequency were only identified in table from 50 most expressed genes: AUC, AUA, GGA, and AGA. The chi-square test shows that there is enough evidence to support the hypothesis that the frequencies of the codons are not homogeneous among the three tables. Therefore, we conclude that there is a change in the codon frequency dependent on the level of gene expression. The next step is the production of three genetic constructs with the green fluorescent protein (GFP) gene, optimized by each of the codon frequency tables in order to observe, *in vivo*, whether there is an improvement in the production of recombinant proteins.
